# Severe Hemorrhagic Cholecystitis in the Absence of Common Predisposing Factors

**DOI:** 10.7759/cureus.72021

**Published:** 2024-10-21

**Authors:** Cristhian Perez Torrico, Seth Williams, Brandon S Radow

**Affiliations:** 1 School of Medicine, West Virginia University, Morgantown, USA; 2 General Surgery, Charleston Area Medical Center, West Virginia University, Charleston, USA

**Keywords:** acute cholecystitis, gallbladder wall thickening, hemorrhagic cholecystitis, open cholecystectomy, subtotal fenestrated cholecystectomy

## Abstract

Hemorrhagic cholecystitis is a rare and potentially life-threatening complication of acute cholecystitis. It lacks specific diagnostic and management guidelines due to its rarity. We present the case of a 70-year-old male with no typical risk factors who developed hemorrhagic cholecystitis. The patient presented with acute right upper quadrant pain radiating to the back and right lower abdomen, accompanied by nausea and fatigue. A CT scan revealed acute hemorrhagic cholecystitis, prompting an emergent open subtotal fenestrated cholecystectomy. Pathology confirmed hemorrhagic cholecystitis without gallstones or masses. The patient recovered uneventfully and was discharged five days after admission.

## Introduction

Hemorrhagic cholecystitis is a rare and potentially life-threatening complication of acute cholecystitis if not recognized early. The condition carries a significant mortality rate, estimated between 15% and 20% based on existing case reports [1]. It has been associated with anticoagulant use, cirrhosis, renal failure, trauma, and malignancy [2,3]. Currently, there are no guidelines in the literature for the diagnosis or management of hemorrhagic cholecystitis due to its rarity. The exact diagnosis can be challenging because of its overlap with other common causes of abdominal pain, nausea, vomiting, and fever. Imaging studies such as ultrasound or computed tomography (CT) can aid in diagnosis [3]. Here, we present the case of a patient who developed hemorrhagic cholecystitis without typical risk factors, including prior symptomatic cholelithiasis, anticoagulant use, cirrhosis, or renal disease.

## Case presentation

**Figure 1:**
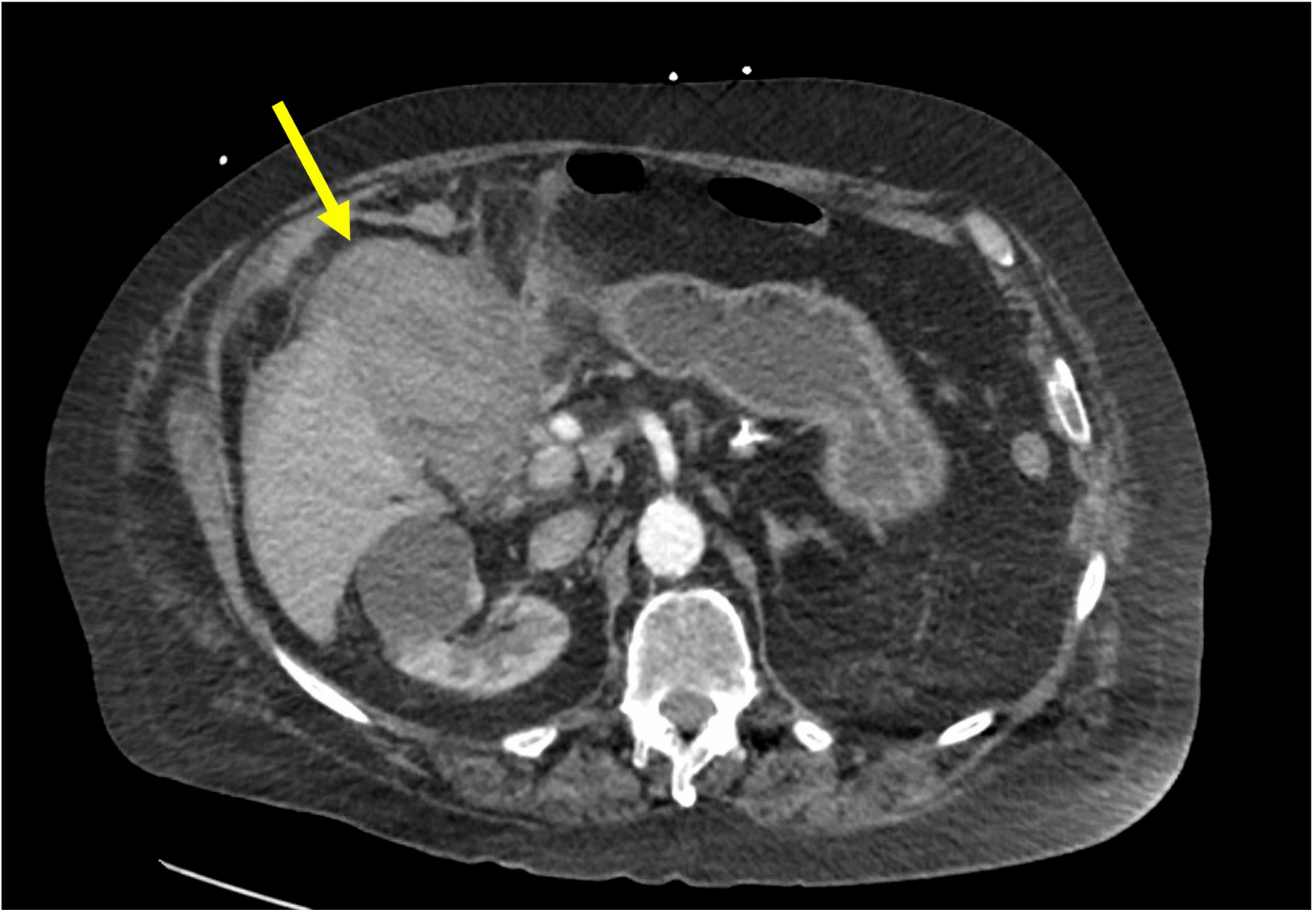
Axial abdominal CT scan showing gallbladder wall thickening and significant distention (yellow arrow).

**Figure 2:**
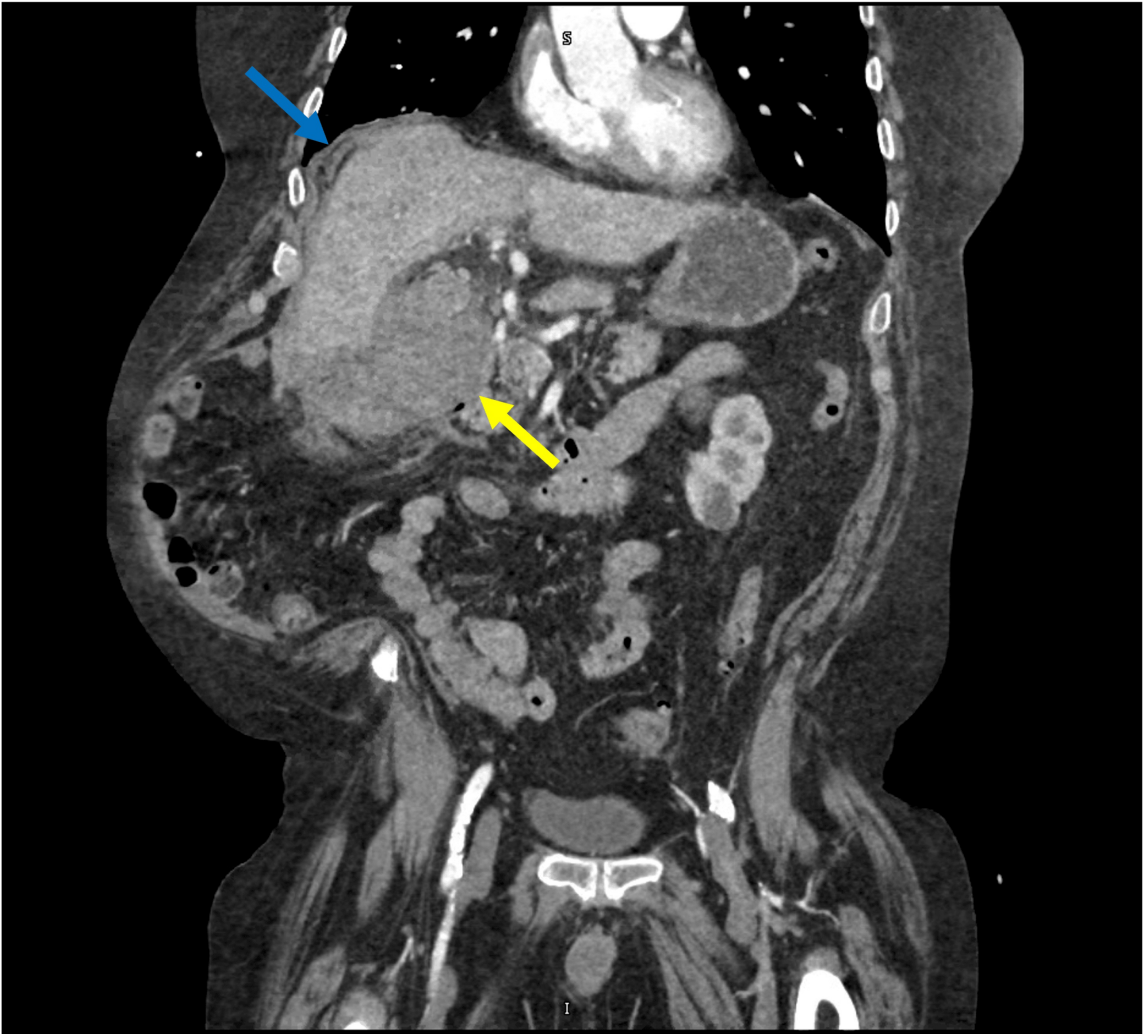
Coronal abdominal and pelvic CT scan showing significant gallbladder distention (yellow arrow) and subhepatic fluid (blue arrow).

A 70-year-old male presented to the emergency department (ED) with acute onset of right upper quadrant pain that started about 12 hours ago while driving. The pain was constant and progressively increased in intensity. Over time, the pain started to radiate to his back and down his right flank to the right lower abdomen. He reported associated nausea and one episode of emesis, lightheadedness, and increased fatigue. He mentioned his last meal was a few hours before the onset of pain and he had decreased appetite since. He denied recent bowel changes. The patient had a past medical history of chronic obstructive pulmonary disease requiring 3 L of supplemental oxygen at home, hypertension, osteoarthritis, and bladder cancer. He was unsure about the specifics of the type of bladder cancer. On presentation, his vital signs were stable with a blood pressure of 110/70 mmHg and a heart rate of 87 beats/minute, and he was afebrile. On abdominal examination, the patient was focally tender in the right upper quadrant with involuntary guarding, positive rebound tenderness, and positive Murphy’s sign. He also experienced pain radiating toward the right lower quadrant. The patient had multiple previous abdominal incisions from a right partial nephrectomy for stone removal, distal pancreatectomy and splenectomy due to concern for malignancy, and an appendectomy. A CT scan from an outlying facility showed a gallbladder consistent with acute hemorrhagic cholecystitis. The CT scan indicated gallbladder wall thickening, significant distention, marked subhepatic fluid, as well as fluid along the right paracolic gutter and pelvis (Figures 1, 2). Laboratory results obtained upon admission are presented in Table 1. The patient denied any use of anticoagulant or antiplatelet medications, chronic kidney disease, or liver disease.

**Table 1:** Laboratory results at hospital admission.

Laboratory Test	Results	Reference range
White blood count	16.3	4.8–10.8 × 10^3^/µL
Hemoglobin	13.1	14.0–16.0 g/dL
Hematocrit	39.7	41.0–53.0%
Platelet count	386	140–450 × 10^3^/µL
Creatinine	1.5	0.7–1.3 mg/dL
Blood urea nitrogen	22	7–25 mg/dL
Total bilirubin	3.1	0.3–1.0 mg/dL
Alkaline phosphatase	369	34–104 U/L
Alanine aminotransferase	224	7–52 U/L
Aspartate aminotransferase	334	13–39 U/L
Prothrombin time	13.3	9.4–12.5 seconds
International normalized ratio	1.17	1.0–1.0

**Figure 3:**
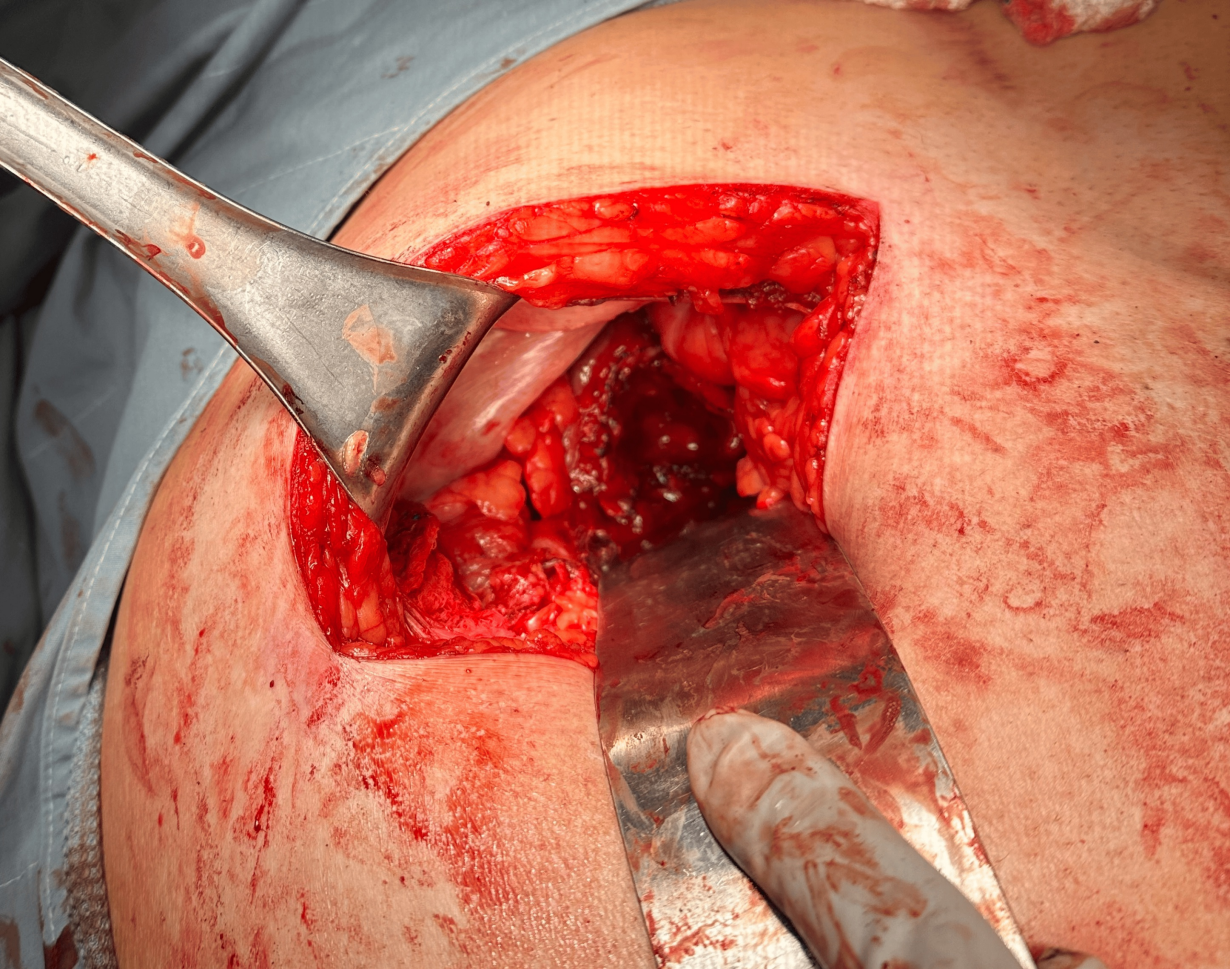
Intraoperative image of the gallbladder fossa.

**Figure 4:**
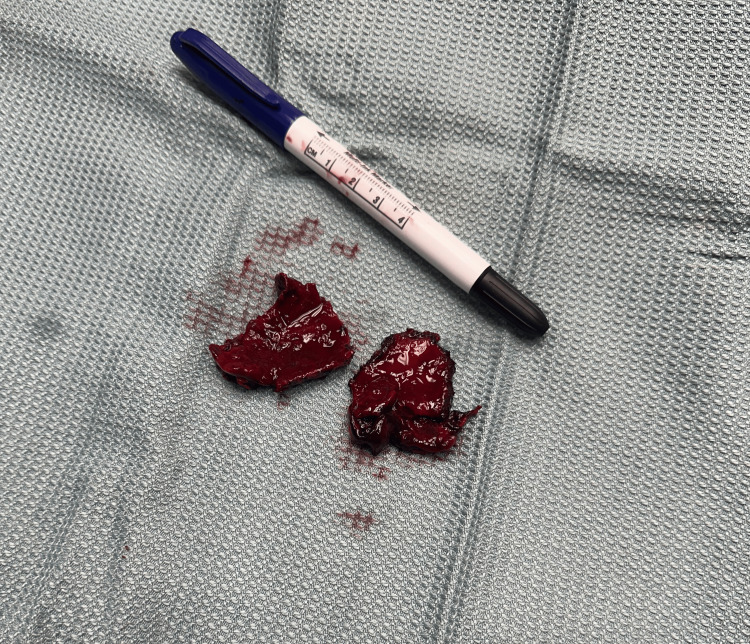
Gross image of the specimen showing a clot within the gallbladder.

The patient underwent an emergent open subtotal fenestrated cholecystectomy with drainage placed in the gallbladder fossa and there was no evidence of ongoing bleeding (Figures 3, 4). The pathology report confirmed severe acute and chronic hemorrhagic cholecystitis and no stones or masses were identified. The patient had an uneventful recovery and was discharged five days after presentation.

## Discussion

Acute cholecystitis accounts for 3-10% of patients presenting with abdominal pain, with hemorrhagic cholecystitis being a rare subset [3]. A literature review of hemorrhagic cholecystitis by Jiang et al. from 1987 to 2017 identified 33 cases, predominantly affecting males (70%) with a mean age of 63 years [3]. Only 21% of patients exhibited signs of hemorrhage, and few experienced hemodynamic instability [3]. Notably, five patients developed hemodynamic instability and required an open cholecystectomy and only two of those patients survived [3]. This is thought to be due in part to delay in diagnosis and patients being at a more advanced stage of the disease.

The exact pathophysiology of hemorrhagic cholecystitis is not well understood, but it is hypothesized to be caused by transmural inflammation leading to gallbladder vessel erosion resulting in hemorrhage into the gallbladder lumen or the abdominal cavity [4]. The use of anticoagulants likely contributes to hemorrhage. Tarazi et al. found that 45% of patients with hemorrhagic cholecystitis were on anticoagulation [5]. Other conditions predisposing patients to bleeding include cirrhosis and kidney disease. In a retrospective study from 2000 to 2021 at two Australian hospitals, Khan Hotak et al. found the incidence of hemorrhagic cholecystitis to be 0.55% among 6,458 who underwent cholecystectomy [4]. Interestingly, only 5.7% of these patients were on anticoagulants, contrasting sharply with earlier case reports [4].

Initial imaging studies for acute cholecystitis typically include right upper quadrant ultrasound and CT scans. However, ultrasound may not be ideal for differentiating between acute cholecystitis and hemorrhagic cholecystitis [3]. Ultrasound and CT have sensitivities of 38.4% and 69.2%, respectively, for detecting hemorrhage in the gallbladder [6]. Therefore, a CT scan is considered the best imaging modality for identifying hemorrhagic cholecystitis. Additionally, magnetic resonance imaging can be used for pregnant women or when ultrasound and CT scan results are inconclusive [4]. Management options for hemorrhagic cholecystitis include open or laparoscopic cholecystectomy and, in some cases, cholecystostomy or nonoperative management with intravenous antibiotics [3,5]. Laparoscopic cholecystectomy is often the preferred treatment with the most favorable outcomes [4,5]. In the presented case, open cholecystectomy was selected due to the potential for hemodynamic instability, severe tenderness, medical comorbidities, and a complex abdominal surgical history to address this rapidly progressive condition. Additionally, a fenestrated subtotal cholecystectomy was performed due to the extent of hematoma found in the abdomen and associated tissue thickness near the infundibulum, making complete gallbladder removal a high risk for associated injury.

## Conclusions

Hemorrhagic cholecystitis is very infrequently encountered but can result in significant morbidity and mortality. Clinicians should maintain a high index of suspicion in patients presenting with symptoms of acute cholecystitis, particularly those on anticoagulants or with hemodynamic instability; however, it is important to note that patients may also present without any predisposing factors. Treatment should be individualized, considering the patient’s overall condition and comorbidities.
